# Mechanical Way To Study Molecular Structure of Pericellular Layer

**DOI:** 10.1021/acsami.3c06341

**Published:** 2023-07-25

**Authors:** Nadezda Makarova, Małgorzata Lekka, Kajangi Gnanachandran, Igor Sokolov

**Affiliations:** † Department of Mechanical Engineering, Tufts University, Medford, Massachusetts 02155, United States; ‡ Department of Biophysical Microstructures, Institute of Nuclear Physics PAN, PL-31342 Kraków, Poland; § Department of Physics, Tufts University, Medford, Massachusetts 02155, United States

**Keywords:** cell mechanics, brush model, urothelial and bladder cancers, pericellular layer, enzymatic treatment, atomic force microscopy (AFM)

## Abstract

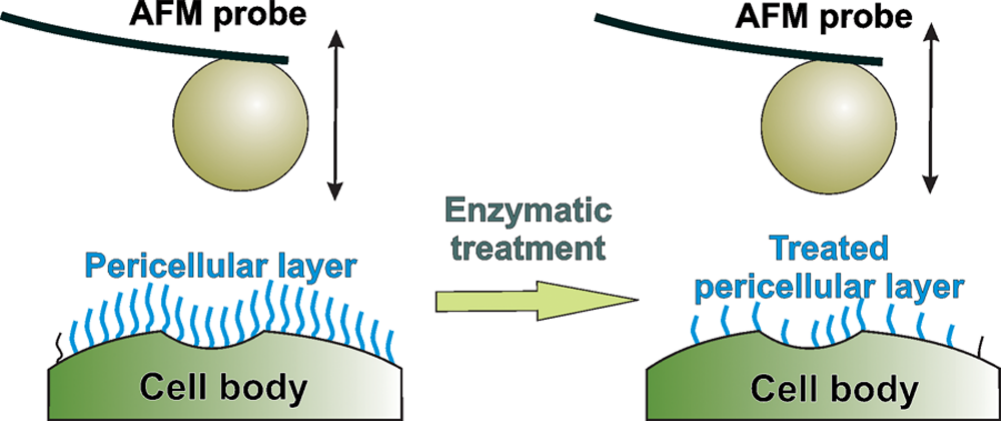

Atomic force microscopy (AFM) has been used to study the mechanical properties of cells, in particular, malignant cells. Softening of various cancer cells compared to their nonmalignant counterparts has been reported for various cell types. However, in most AFM studies, the pericellular layer was ignored. As was shown, it could substantially change the measured cell rigidity and miss important information on the physical properties of the pericellular layer. Here we take into account the pericellular layer by using the brush model to do the AFM indentation study of bladder epithelial bladder nonmalignant (HCV29) and cancerous (TCCSUP) cells. It allows us to measure not only the quasistatic Young’s modulus of the cell body but also the physical properties of the pericellular layer (the equilibrium length and grafting density). We found that the inner pericellular brush was longer for cancer cells, but its grafting density was similar to that found for nonmalignant cells. The outer brush was much shorter and less dense for cancer cells. Furthermore, we demonstrate a method to convert the obtained physical properties of the pericellular layer into biochemical language better known to the cell biology community. It is done by using heparinase I and neuraminidase enzymatic treatments that remove specific molecular parts of the pericellular layer. The presented here approach can also be used to decipher the molecular composition of not only pericellular but also other molecular layers.

## Introduction

Various techniques used to identify the mechanical properties of single cells or even tissues have shown that cell mechanics correlates well with cancer progression.^1,2^ By delivering quantitative measures of cancer-related changes, these techniques shed light on our understanding of cancer initiation, progression, dissemination, and metastasis. Atomic force microscopy (AFM^3^) is a versatile technique capable of recording distinct signals related to mechanical^4,5^ or rheological^6,7^ or adhesive^8^ properties of biological samples. Such broad AFM functionality is a strong driving force for introducing AFM to clinical practice as a fundamentally new type of nonlabeling biomarker for the diagnostics and/or prognosis of cancer and probably other diseases.

It has been demonstrated that cancer cells adapt and change in response to external stimuli or mechanical properties of the extracellular matrix (ECM), changing to more invasive phenotypes.^9,10^ Cell interactions with ECM components, such as proteins or proteoglycans, regulate cellular functions, including maintaining cell shape, adhesion, migration, proliferation, polarity, differentiation, and apoptosis.^9,11^ In pathological conditions, increased synthesis of specific ECM components can contribute to cancer growth and progression.^12,13^ The cellular response depends on the nature of the adhesion receptors, i.e., integrins and cadherins. The change in microenvironment stiffness can initiate alterations in their expression. Integrins are linked to the actin molecules of the cytoskeleton. Thus, changes in their expression contribute to cell deformability. Findings show that most cancer cells are characterized by larger deformability, which is believed to be one of the causes of altered cell adhesive and migratory properties.^14,15^ The altered adhesive properties of cancer cells have been a target for numerous research attempts to identify molecules for inhibiting cancer invasion due to their accessibility from the cell exterior (i.e., from the ECM side). The cell surface contains numerous adhesive molecules, such as integrins^16^ or cadherins.^17^ These molecules, together with proteoglycans attached to the pericellular membrane^18^ like syndecans,^19^ form a layer called glycocalyx.^20^

The alteration of glycocalyx on malignant cells was studied with the AFM technique for cervical cancer.^21^ The AFM indentation data were processed through the brush model,^21,22^ the approach that allows the separation of the mechanical properties of the cell body and its pericellular layer. It has been shown that AFM can detect both the presence of glycocalyx and corrugation of the pericellular membrane (like microridges and microvilli^23^). Despite the relative complexity of the model, it was shown that it is rather robust; i.e., it allows for rather precise separation of the mechanical deformation of the cell body from the physical properties of the pericellular brush layer.^24,25^ For example, it was shown that a possible error in the definition of the quasistatic Young’s modulus of the cell body does not exceed 4% due to the model and experimental uncertainties. To amplify, the brush model allows us to derive the quasistatic Young’s modulus of the cell body accompanied by information on the biophysical properties of the pericellular brush layer in a robust self-consistent way.

The present study focuses on human bladder epithelial cancer cells. Bladder cancer (BC) is one of the most common cancers worldwide. It is characterized by high incidence and mortality rates.^26^ Although this cancer is effectively treated if captured early, a 50–80% recurrence rate requires continuous monitoring of patients for recurrence and/or progression to a more advanced stage (once every 3–6–12 months, which is the current practice for patients with non-muscle-invasive tumors (75% of newly diagnosed bladder cancers). The monitoring includes invasive optical bladder examination (cystoscopy) and possible tumor resection for pathology examination. The requirement for frequent cystoscopy makes BC the most expensive cancer per patient to diagnose, monitor, and treat. Numerous global authorities recognize it as a major health issue incurring a significant burden on healthcare systems.^27^

The bladder is characterized by a large degree of mechanical flexibility linked with its physiological role^28^ and the greater adaptability of these cells to altered microenvironments.^29,30^ Despite that, it was possible to distinguish cancer cells from nonmalignant or benign cells based on their mechanical properties.^5,31^ Changes in the mechanical properties of epithelial cells can be related to actin cytoskeleton organization. The alterations in actin organization might be related to the adhesive properties of the bladder cells. Immunohistochemical analysis has already shown that normal human urothelium expresses integrins built of such subunits as α3, αV, β1, and β4.^29,32^ In parallel studies, a strong correlation has been found between the metastatic activity of cells and glycocalyx composition in cancer cells, including sialylation, fucosylation, O-glycan truncation, and N- and O-linked glycan branching.^20^ The biological functions of glycocalyx-associated molecules lie in the ability to interact with various ligands modulating the interaction of cells with ECM; thus, they can be involved in such processes as epithelial–mesenchymal transition (EMT) and carcinogenesis.^33^

Atomic force microscopy allows obtaining of high lateral resolution of mechanical properties of the pericellular layer. However, it is frequently difficult to understand the biological significance of this information. Therefore, it is instrumental to find a way to translate the obtained mechanical information into biomolecular data, which is the ground for biological and medical understanding of the significance of the pericellular layer. Here we suggest the use of enzymatic treatment to remove particular molecular parts of the pericellular coat and to correlate it with the AFM measured mechanical properties of the pericellular layer. Specifically, we aim to investigate sialic acid (SA) and heparan sulfate (HS) residues of the glycocalyx proteoglycans. HS is one of the proteoglycan family encompassing polysaccharide-based chains bound to such proteins as glypicans or syndecans.^34,35^ The expression of HS can be altered after the transformation from noninvasive to invasive states.^36^ The abnormally high presence of SA residues is also considered a distinctive feature associated with malignancy and the invasiveness of cancers.^37^ Both proteoglycans and glycans, present on the cell surface, could be used as cancer identification and treatment targets. Due to their complexity and heterogeneity, methods for analyzing their properties in the cell surface context are strongly needed.

The presence of HS chains and SA residues is evaluated here using AFM by cleaving the residues with specific enzymes. We use heparinase I (hep) and neuraminidase (neu) enzymes to selectively remove HS and SA residues from the cell surface. By analyzing the indentation force curves with the help of the brush model, one can obtain the parameter of the pericellular brush layer, which includes glycocalyx. Here we found that cancer and nonmalignant HCV29 cells possess two brush layers (inner and outer). The inner brush in nonmalignant HCV29 cells was shorter than that of transitional cell carcinoma TCCSUP cells but of the same effective molecular density. The accompanying outer cell brush was larger and denser than in the case of cancer cells. Furthermore, we found that changes in the cellular brush depend on the type of enzyme applied. A substantial change in the pericellular brush layer observed after enzymatic treatment indicates the presence of either HS chains or SA residues. The mechanical properties of the cell body or the inner part of the pericellular brush layer were not changed after hep treatment, neither in HCV29 nor in TCCSUP bladder cells. In the case of neu treatment, the stiffening was observed only after neu treatment in TCCSUP cells and not in nonmalignant HCV29 cells. Notably, a substantial difference in the rigidity of the cell body agrees with the previous observations showing that nonmalignant bladder cancer cells were considerably more rigid.^5^

## Results and Discussion

The glycocalyx is a dense network of glycans bound to glycoproteins, glycolipids, and proteoglycans, playing a crucial role in the interactions of cancer cells with the microenvironment.^38^ Proteoglycans are frequently dysregulated during cancer progression,^39^ and therefore, they possess the potential to be a target for inhibiting cancer invasiveness, already demonstrated for heparan sulfates.^40^ Altered sialylation, a hallmark of cancer progression, is critical for glycans as the addition of sialic acids at the terminal end of glycans changes a charge bearing by these molecules (sialic acid is the only sugar that carries a (negative) charge^41^). As the sialylation of molecules responsible for cell adhesion can increase the metastatic potential of many cancers,^42^ their desialylation may inhibit the dissemination of cancer cells.^43^ Consequently, the structure and function of various glycans, glycoproteins, or glycolipids are altered.^44^ Thus, it is plausible to expect that cancer cells show specific alterations in their glycocalyx.

Besides comparing nonmalignant and malignant (cancerous) bladder epithelial cells, our study includes a quantitative description of glycocalyx properties before and after the enzymatic cleavage of proteoglycans and terminal sialic acid residues by heparinase I and neuraminidase. Such a treatment cleaves the glycocalyx containing those residues, thereby changing the mechanical properties of the pericellular brush layer. AFM is a suitable method for examining the physical properties of cell glycocalyx.^21,24,25,45^

**Figure 1:**
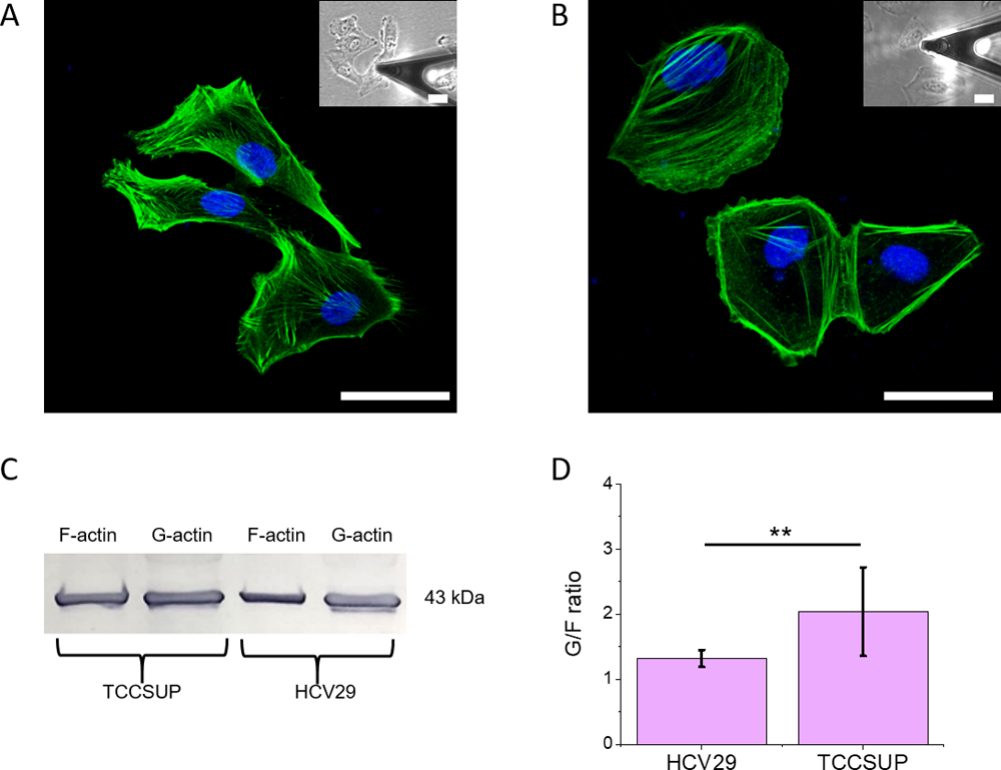
Actin content and organization in bladder cancer cells. (A, B) Fluorescent (confocal) images of nonmalignant HCV29 (A) and cancerous TCCSUP (B) cells. Actin filaments were visualized through F-actin staining by phalloidin conjugated with fluorescent Alexa Fluor 488, while cell nuclei were stained with Hoechst 33342 dyes. Insets: images of cells on a Petri dish surface measured by AFM. Scale bars, 20 μm. (C, D) G/F actin ratio determined for HCV29 and TCCSUP bladder cancer cells. (C) Exemplary results from Western blot show total G- and F-actin expression. The used antibody (against β-actin) targets all known actin isoforms with a molecular mass of 43 kDa (globular actin and fibrous actin). (D) The mean ± SD of the G/F ratio was determined for cells (*n* = 4 independent experiments; statistical significance was calculated using an unpaired *t* test at the significance level of 0.05).

First, we study the mechanical properties of the cell body. The link between the mechanical properties of cells and the actin cytoskeleton has been reported in various research.^4,46,47^ Thus, we started the experiments to characterize the actin content and filament organization in nonmalignant HCV29 and transitional cell carcinoma TCCSUP cells. The actin filaments were stained with fluorescent Alexa Fluor 488 dye conjugated with phalloidin. Phalloidin binds specifically to F-actin, a polymerized form of actin, at the binding site between F-actin subunits.^48^ Therefore, this dye is widely applied to visualize filamentous forms of actin. The cell nucleus was labeled with the Hoechst 33342 dye. Confocal images showed that both cell lines are morphologically different (Figure 1A,B). HCV29 cells are elongated, revealing spindle-like morphology with a well-developed actin cytoskeleton, which showed pronounced thick actin filaments being actin bundles composed of, probably, single stress fibers. TCCSUP displays a rather cubic (more rounded) shape with visible thick actin bundles.

**Figure 2:**
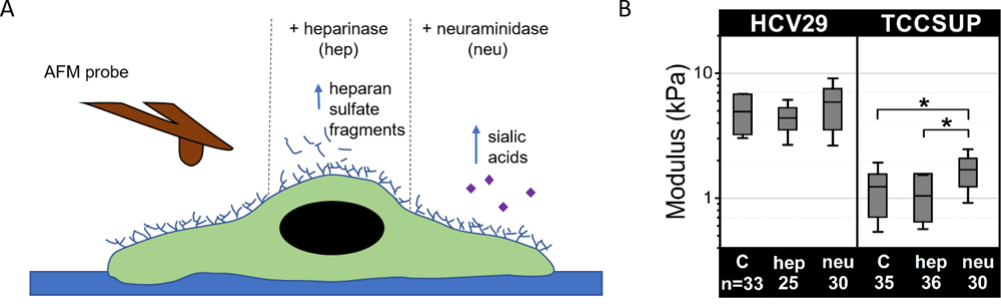
Probing brush on the surface of cancer cells. (A) The idea of AFM measurements showing a cellular brush before and after the enzymatic treatment removing either heparan sulfate chains (heparinase I, hep) or sialic acids residues (neuraminidase, neu) from glycocalyx. (B) The elastic properties of nontreated and enzyme-treated cells were quantified by the quasistatic Young’s modulus. Boxplot represents the mean and the 25th and 75th percentile range of data, with each whisker as one standard deviation (*p < 0.05, Kruskal–Wallis ANOVA).

Knowing from our previous studies^4,7,22,49^ that mechanical properties can also be related to the actin content, we quantify the expression of two actin forms, i.e., F-actin and G-actin, present inside the cell (Figure 1 C,D). Its value close to (and above) 1 indicates that majority of known actin isoforms with a molecular mass of 43 kDa are polymerized, while its value close to 0 indicates the opposite; i.e., they are in monomeric G-actin form. The results showed that the F/G actin ratio is smaller in nonmalignant HCV29 than for transitional cell carcinoma TCCSUP cells, which denotes the higher level of monomeric G-actin in these cells. The mechanical properties of the cell body and the physical properties of the pericellular brush were measured using AFM. (Figure 2A). The examples of the analyzed force curves are shown in Supporting Information Figures S1 and S2. The results for the quasistatic Young’s modules are shown in Figure 2B. The cells were treated with two enzymes: neuraminidase (neu) and heparinase I (hep). Each of them acts differently on cell glycocalyx. Neuraminidase cleaves terminal sialic acid residues in glycans.^50^ As a result, the shortening of glycan structure is observed (Supporting Information Figure S3A, exemplary branched sialylated N-glycans cleaved by neuraminidase). Heparinase I removes heparan sulfate chains constituting the proteoglycans.^34^ In such a case, larger fragments of heparan sulfate chains are cleaved (Supporting Information Figure S3B, showing illustrative heparan sulfate attached to syndecans) compared to neuraminidase cleavage.

The mechanical properties of bladder cancer cells have already been measured.^5,29,49^ The results showed a larger deformability of cancerous cells in relation to the control, nonmalignant cells. However, in those studies, the AFM measurements and data analysis were conducted without considering the presence of the pericellular brush layer (which includes the glycocalyx) surrounding cells. As was shown,^24,25^ the mechanical contribution of the pericellular layer is significant; ignoring the pericellular brush layer results in the dependence of Young’s modulus of the cell on the indentation depth. Thus, it does not allow us to compare the mechanical properties of cells directly and to measure the absolute values of the Young’s modulus. The brush model used in the present work allows for deriving the quasistatic Young’s modulus of the cell body in a robust self-consistent way, in which the modulus does not depend (or just weakly depends) on the indentation depth. Thus, the present observation of a significant softness of the malignant compared to the nonmalignant cells is a rather nontrivial and important result.

Figure 2B shows the results for determining the quasistatic Young’s modulus of cells. Both nonmalignant and malignant (cancer) cells were studied before and after the treatment with the two enzymes described above. One can see that the cancer TCCSUP cells are softer than nonmalignant HCV29 cells. This difference is in agreement with the observed changes in the actin cytoskeleton density that included both the 2D spatial organization of actin filaments (measured optically) and the measured higher amount of monomeric G-actin in cancer cells (see Figure 1). Furthermore, the results shown in Figure 2B demonstrate that nonmalignant HCV29 cells are insensitive to enzyme treatment as the quasistatic Young’s modulus remained at the same level of 5 kPa, i.e., 4.93 ± 1.90 (*n* = 33), 4.39 ± 1.72 (*n* = 25), 5.87 ± 3.23 kPa (*n* = 30) for nontreated, hep- and neu-treated cells, respectively. In the case of cancerous TCCSUP cells, the quasistatic Young’s modulus remained at the same level as nontreated cells after removing heparan sulfate fragments; however, removing sialic acid residues induced some stiffening of cells. The quasistatic Young’s moduli were 1.23 ± 0.70 kPa (*n* = 35), 1.05 ± 0.48 kPa (*n* = 35), and 1.69 ± 0.77 kPa (*n* = 30), respectively.

**Figure 3:**
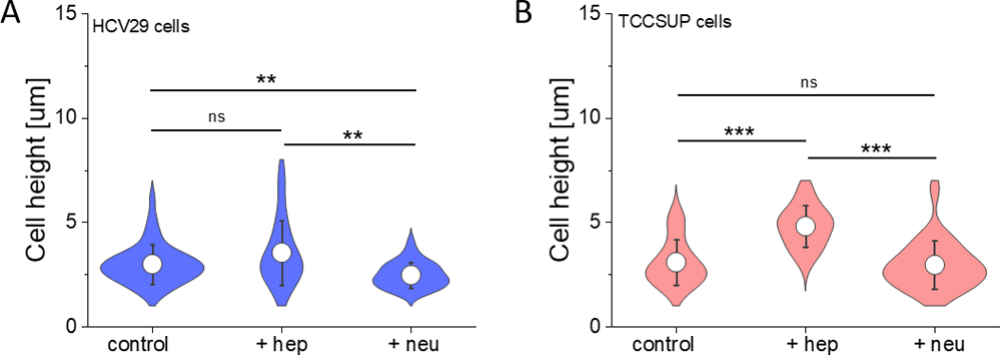
Height of nontreated and enzyme-treated cells: (A) nonmalignant HCV29 cells and (B) transitional cell carcinoma TCCSUP cells. Violon plot represents the distribution of data with a mean (white dot) and SD calculated from 20 to 50 cells depending on the conditions (statistical significance: ***p < 0.001, **p < 0.01, ns = not statistically significant, Kruskal–Wallis ANOVA).

Considering the mechanisms of hep and neu cleavage, we expected changes in the cell height as different fragments of glycocalyx were removed from the cell surface (the method of measuring the cell height determination is described in the Supporting Information, Figure S7). The effect was cell- and enzyme-dependent (Figure 3).

The height of HCV29 cells (2.99 ± 0.94 μm, *n* = 33 cells) remained unaltered upon hep treatment (3.55 ± 1.54 μm, *n* = 25 cells, *p* = 0.2585), but it decreased when neu was applied to the cells (2.47 ± 0.61 μm, *n* = 30 cells, *p* = 0.0064). In the case of cancer TCCSUP cells, the nontreated cell (control) height was 3.09 ± 1.09 μm (*n* = 35 cells). It increased for cells treated with hep to 4.81 ± 0.99 μm (*n* = 36 cells, *p* > 0.001) and remained similar to control cells after neu treatment (2.96 ± 1.16 μm, *n* = 30 cells, *p* = 0.8595). Changes in cell height indicate remodeling of the cell cytoskeleton, which might contribute to cell mechanics. However, the lack of direct correlation with the cell mechanical properties indicates that, most probably, such remodeling involves short actin filaments, and stress fibers remained responsible for the mechanical properties of cells (their organization remained unaltered, Supporting Information Figure S4).

Hep treatment applied to nonmalignant HCV29 cells did not affect the height and mechanical properties of the cells. In the case of cancerous TCCSUP cells, hep treatment did not affect the cell mechanics but the cell height increased. Heparan sulfates are attached to proteins that are neither directly nor indirectly attached to actin filaments.^51^ Thus, we can speculate that the hep cleavage removes only fragments of heparan sulfates, leaving the actin cytoskeleton unaffected. The increase in cell height may be correlated with the altered osmolarity of the environment manifested as the cell diameter increase.^52^

Neu treatment (i.e., desialylation of cells) did not affect the mechanical properties of nonmalignant HCV29 cells, although the cell height decreased. Glycans containing terminal sialic acid residues are attached to the cell adhesion molecules, such as integrins or cadherins. Thus, their desialylation may affect direct or indirect attachment to the actin cytoskeleton. Lack of rigidity changes in HCV29 cells can be linked to a smaller amount of sialic acids in these cells than in bladder cancer cells.^53^ In parallel, the stiffening of TCCSUP cells after neu treatment could indicate a strong local reorganization of the actin cytoskeleton. For these cells, the cell height remained unaltered. In addition, the epi-fluorescent images of the actin cytoskeleton did not show significant changes, which indicates that cytoskeleton remodeling is not the leading cause of TCCSUP cells stiffening (Supporting Information Figure S4). Negatively charged sialic acid residues provide charge repulsion and prevent unwanted cellular interactions.^54^ Thus, removing these residues changes the properties of the glycocalyx, probably leading to its partial collapse, as has already been seen for endothelial cells.^55^ Such collapse can form a stiffer shell around the cell. The results presented in Figure 6B,D (see later in the paper) vote in favor of this conclusion. One can see that the density of the outer brush increases substantially after the treatment, although the size does not.

**Figure 4:**
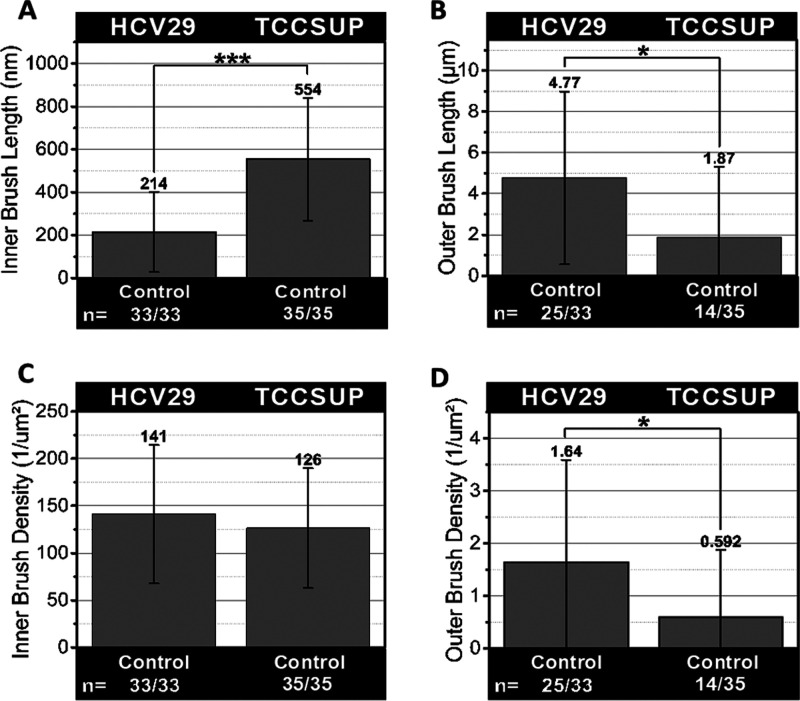
Probing the pericellular brush on the surface of bladder cells without enzymatic treatment. (A, B) Length and (C, D) corresponding density of the surface brush. Data are expressed as the mean ± standard deviation for 33 cells (***p < 0.001, *p < 0.05).

The pericellular brush is composed of two main parts. One is the glycans that can be considered as a layer of polymeric chains grafted to the cell membrane, while the second part is the membrane protrusions that can be considered as random asperities. Deformation of both parts can be described with the help of exponential force dependence. Therefore, we used the Alexander DeGenne model (exponential version) for polymer brush to approximate the pericellular brush^56^ (eqs 6 and 7). Later, using an example of human cervical epithelial cells,^21^ it was shown that the pericellular layer of cancer cells could be characterized by a brush with two characteristic sizes (0.45 and 2.6 μm long). Using guinea pig fibroblasts,^57^ it was demonstrated that the pericellular layer might consist of a shorter and relatively rigid inner part, which comprises corrugations of the pericellular membrane, and an outer part comprised mainly of oligosaccharides and glycoproteins (what is traditionally called glycocalyx). Here the brush model was applied to obtain not only the mechanical properties of the cell body but also the physical characteristics of the pericellular brush layer, its thickness (length), and effective surface density (Figure 4). It should be noted that multiple cells and force curves demonstrate double brush behavior (see Supporting Information Figures S1 and S2). We considered the properties of the inner and outer brush layers separately.

**Figure 5:**
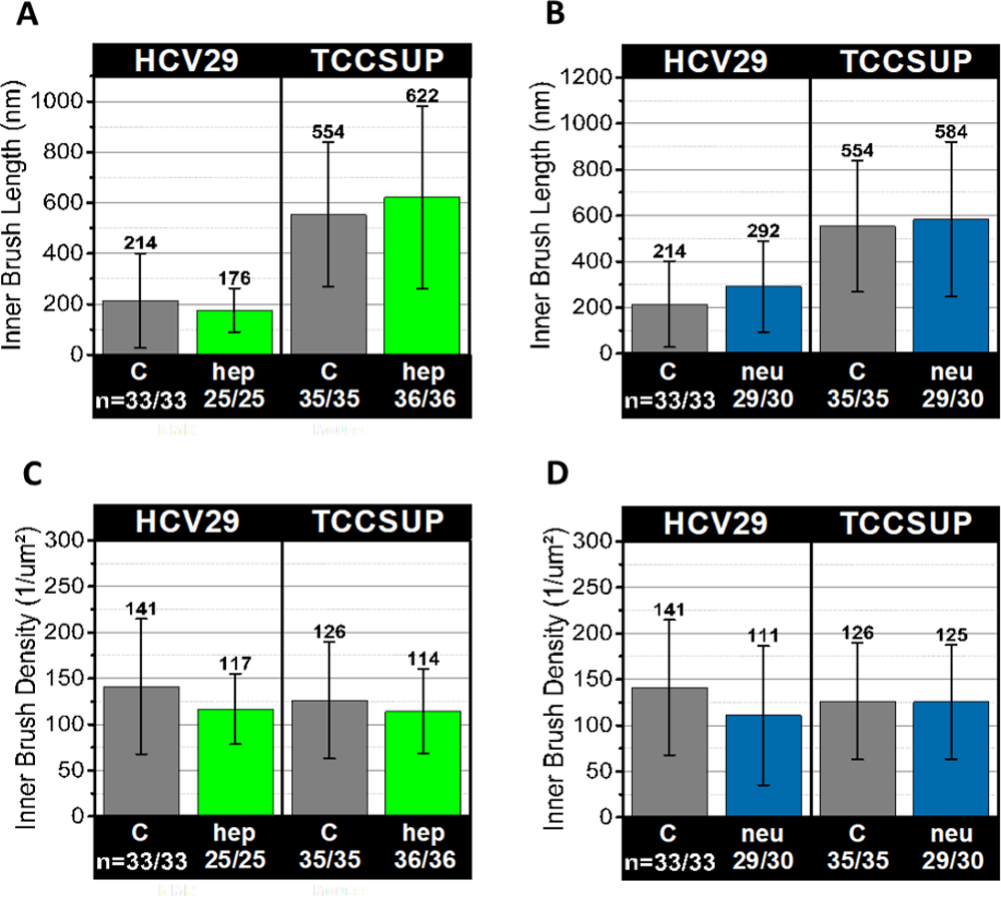
Length and density of the inner brush on the surface of bladder epithelial cells. The results for nonmalignant and cancer cells before and after hep (A, C) and neu (B, D) treatment. The mean values and one standard deviation are shown; *n* denotes the number of measured cells (statistically significant differences were not found).

The results show that the inner brush is shorter in nonmalignant HCV29 cells (Figure 4A) than in cancer cells (210 ± 190 nm for nonmalignant HCV29 cells versus 550 ± 290 nm for cancer TCCSUP cells). The corresponding brush density (Figure 5C) remained at a similar level of ∼0.15 μm^–2^. The outer brush length (Figure 4B) for HCV29 cells was also larger (47 ± 42 μm, *n* = 33 cells) than for TCCSUP (18 ± 34 μm, *n* = 33 cells), showing only weak statistical significance (*p* < 0.05). The corresponding brush density (Figure 4D) was 1.64 ± 1.95 μm^–2^ and 0.59 ± 1.28 μm^–2^. This is statistically significant at *p* < 0.05. Overall, we can conclude that HCV29 possess a shorter inner brush than cancer TCCSUP cells, but their density remains similar. The outer brush of cancer cells is almost 2 times smaller and 3 times less dense. Enzyme treatment did not affect the inner brush in these cells, indicating no cleavage sites for enzymes located within the inner brush (Figure 5).

The enzyme treatments showed no significant changes in the brush length of HCV29 cells from 210 ± 190 nm (*n* = 33) for control and 170 ± 90 nm (*n* = 25) for hep and 290 ± 200 nm (*n* = 30) for neu. The corresponding brush density also showed no changes, 140 ± 75 μm^–2^ (*n* = 33) for control, 120 ± 38 μm^–2^ (*n* = 25) for hep, and 110 ± 76 (*n* = 30) for neu. For TCCSUP cells, no significant changes in brush length were also observed for control and enzymatic-treated cells, i.e., 550 ± 290 (*n* = 35, control), 620 ± 360 (*n* = 35, hep), and 580 ± 340 nm (*n* = 30, neu). The corresponding inner brush density did not change too, 130 ± 64 μm^–2^ (*n* = 35, control), 120 ± 46 μm^–2^ (*n* = 35, hep), and 130 ± 62 μm^–2^ (*n* = 30, neu). According to ref (57), it is plausible to state that the inner brush is presumably just the corrugation of the pericellular membrane. Therefore, enzymatic treatment of the glycocalyx does not influence it. Larger membrane corrugations already seen in melanoma cells explain the longer inner brush in cancer cells.^58^

**Figure 6:**
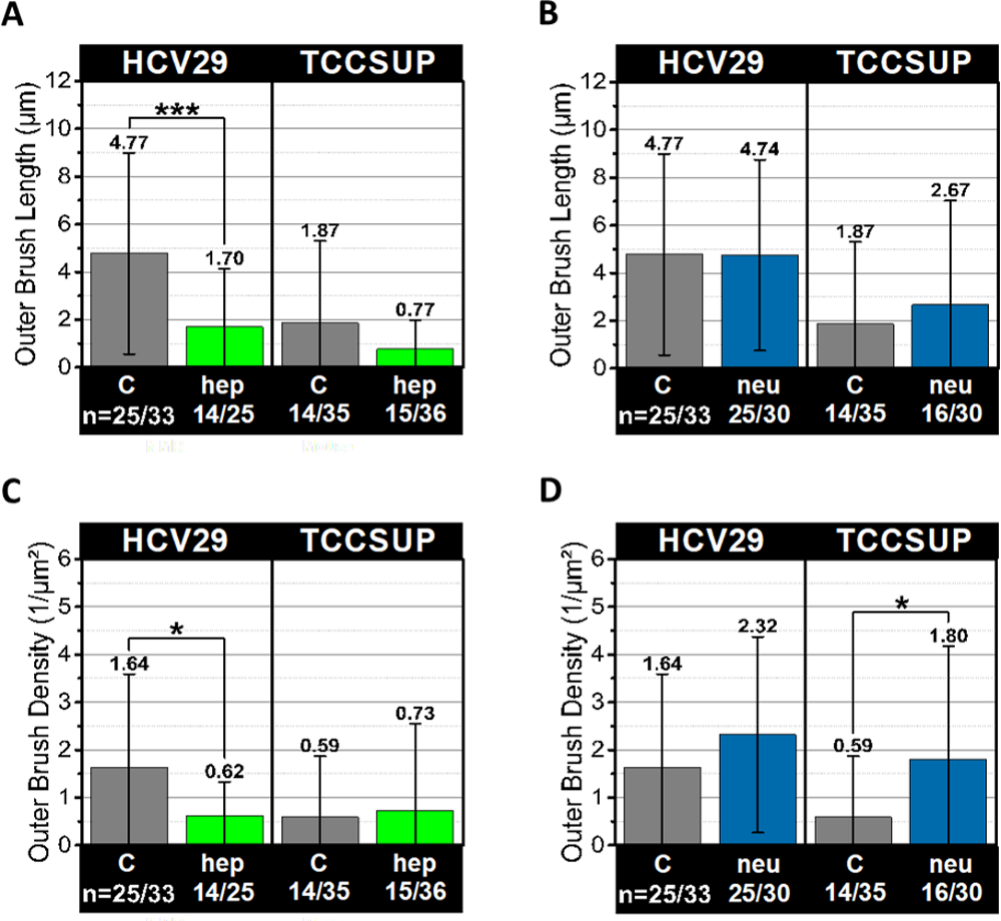
Length and density of the outer brush on the surface of bladder epithelial cells. The results for nonmalignant and cancer cells before and after hep (A, C) and neu (B, D) treatment. The mean values and one standard deviation are shown; *n* denotes the number of measured cells (*p < 0.05, ***p < 0.001).

The analysis of the outer (longer) brush shows that this brush is longer for nonmalignant HCV29 than for cancerous TCCSUP cells (Figure 6).

The enzyme treatment brought similar results for HCV29 cells; i.e., after applying heparinase I, the outer brush became shorter by more than 2 times (from 47 ± 42 μm (*n* = 33 cells) to 17 ± 24 μm (*n* = 25), with density decreasing from 1.60 ± 2.0 μm^–2^ (*n* = 33) to 0.60 ± 0.70 μm^–2^ (*n* = 25). The action of neuraminidase showed no significant effect on length or density in HCV29 cells (47 ± 40 μm and 2.3 ± 2.1 μm^–2^ (*n* = 30). For TCCSUP cells treated with either heparinase or neuraminidase, no significant changes in the outer brush length were detected. The only significant change is an increase in the outer brush density, i.e., 0.59 ± 1.3 μm^–2^ (*n* = 35, nontreated cells) to 1.8 ± 2.4 μm^–2^ (*n* = 30, neu-treated cells).

**Figure 7:**
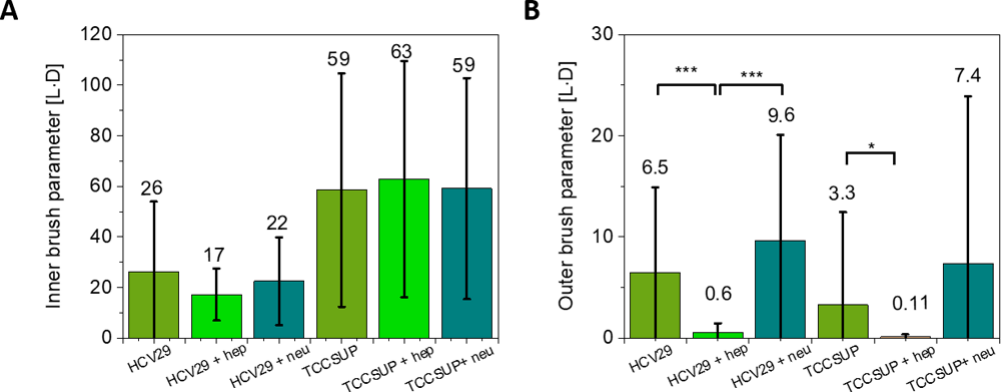
Total size of the inner (A) and outer (B) brushes for nonmalignant and cancer bladder cells before and after the enzymatic treatments. The total size is characterized by multiplying the brush length and density for each force curve and then averaging. Data represent the mean ± standard deviation.

To understand the effect of the enzyme on the cleavage of the glycocalyx part of the pericellular brush layer, it is instructional to combine both brush parameters, the length *L* and the grafting density *N*, into its multiplication, *L × N*. This new parameter characterizes the “total length” of all brush molecules per unit area. The results for the inner and outer brushes are shown in Figure 7.

No statistically significant change in the inner brush mainly reflects the absence of changes in the inner brush as seen in Figure 7A. Moreover, the inner brush was unaffected by the enzyme treatment, which indicates the conclusion that binding/cleavage sites for both enzymes applied here are not present in the inner brush. The inner brush parameter correlates with an increased brush length observed in cancer cells (the density of the inner brush is similar in both studied bladder cells). Highly pronounced changes were observed for the outer brush (Figure 7B). The total brush size was larger for nontreated HCV29 cells than for cancerous TCCSUP cells. The enzyme treatment alters the brush size. The total size of the outer brush decreased from 6.5 to 0.6 (11 times) and from 3.3 to 0.11 (30 times) for HCV29 and TCCSUP cells, respectively. It implies that glycocalyx on nonmalignant and cancerous cells has heparan sulfate as its major part. The brush size after neu treatment is not significantly different from the control, nontreated cells, neither for nonmalignant HCV29 nor for cancer TCCSUP cells. This agrees with the mechanism of sialic acid removal. The enzyme removes only residual sialic acids attached to the terminal ends of the surface glycans. Sialic acid is a negatively charged sugar molecule that consists of 9 carbon atoms (theoretical calculations predicted a very weak C–C bond with a bond length of around 1.8 Å.^59^ Thus, a single molecule has a size of ∼1–2 nm. This size is beyond the detection of our measurements because glycocalyx is a sterically stabilized object; therefore, disbalance in the charges can substantially change the overall architecture of long polysaccharide molecules comprising the brush. As one can see from Figure 6D, the effective grafting brush density increases substantially after the removal of sialic acid molecules. Since the total number of molecules stays the same, the change should be only in the mechanical properties of this brush layer. It becomes more rigid. Presumably, the long polysaccharide molecules stick together by forming a more rigid structure. It is schematically presented in the summary figure (see Figure 9).

**Figure 8:**
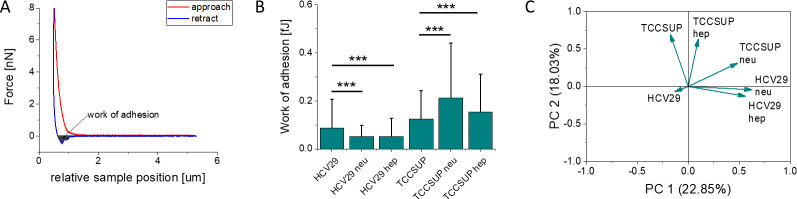
(A) Exemplary force curve recorded during indenting a cell with an AFM probe. The gray area marked within the adhesive part of the retraction curve is the measure of the work of adhesion, i.e., the work needed to detach the AFM probe from the cell surface. (B) Work of adhesion determined for both enzymatically treated and nontreated (control) cells. The mean values and standard deviations are shown. (C) PCA score plot showing a significant separation of data sets collected for enzymatically treated and nontreated (control) cancerous TCCSUP cells. A weak separation of the data sets is seen for enzymatically treated nonmalignant HCV29 cells. Nontreated (control) HCV29 cells separate significantly from both cancerous TCCSUP and enzymatically treated HCV29 cells.

During the AFM measurements, the pull-off force, which we can call adhesion, was observed during retraction of the AFM probe from the cell surface. We quantify this adhesive part of the force curve by calculating the work needed to detach the AFM probe from the cell surface (Figure 8A).

The results show a lower work of adhesion for nonmalignant HCV29 than for cancerous TCCSUP cells (Figure 8B). Because of the nonsymmetric character of histograms (see Supporting Information Figures S5 and S6) and large errors, we confirm distinct adhesive properties of cell surfaces by applying principal component analysis (PCA) to the obtained data. It clearly separated the work of adhesion for cancer cells, denoting nontreated, 0.12 ± 0.12 fJ, and enzyme-treated, 0.21 ± 0.23 and 0.15 ± 0.16 fJ for hep and neu treated TCCSUP cells. Interestingly, cells treated with hep I had different adhesive properties than cells treated with neu. For nonmalignant HCV29 cells, there is a clear separation between nontreated (0.087 ± 0.120 fJ) and enzyme-treated cells (0.051 ± 0.047 fJ and 0.052 ± 0.077 fJ for hep- and neu-treated cells, respectively). The separation between hep- and neu-treated cells is very small, indicating similar adhesive properties of cells before and after the enzyme treatment. Heparan sulfate and sialic acid residue cleavage change the adhesive properties of both cell types by altering the structure, charge, and conformation of the glycocalyx. Hemispherical silicon nitride AFM probes adhere differently to nontreated cells, revealing that nonmalignant HCV29 cells are less adhesive than cancerous TCCSUP cells. The enzymatic treatment decreased and increased the adhesiveness of nonmalignant and cancer cells, respectively. Since no specific interactions are expected, the adhesive properties are defined by the area of contact between the AFM probe and sample surface. The differences in the contact area could be explained by the roughness of the pericellular membrane, which is essentially the inner brush. Furthermore, a denser inner brush should definitely contribute toward higher hydrodynamic drag force, resisting the AFM probe from pulling out of the contact. Comparing the amount of the inner brush shown in Figure 8A with the work of adhesion shown in Figure 8B, one can see a rather good correlation. Thus, the observed difference may indeed be explained by the difference in the inner brush. Finally, the softer malignant cells should comply more under the same force and, therefore, allow for the development of a larger contact area.

**Figure 9:**
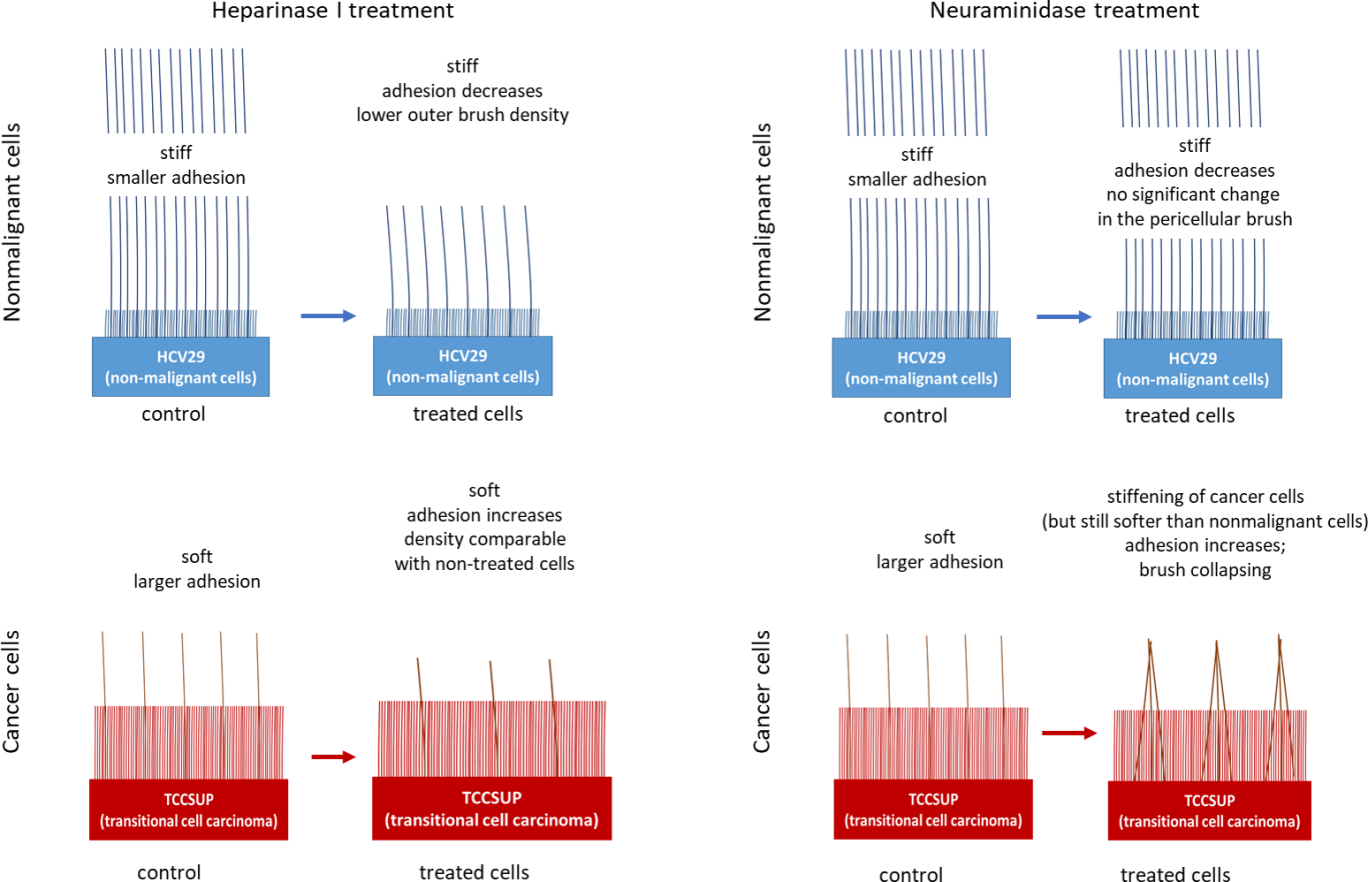
Differences in the pericellular brush structure in nonmalignant and malignant (cancerous) bladder cells in terms of the cell mechanical and adhesive properties and their changes due to enzyme treatment.

Based on the obtained results, we present the differences in the glycocalyx structure in nonmalignant and cancerous cells in a graphical way (Figure 9). This figure also shows the pericellular brush evolution during enzymatic treatment in terms of the cell’s mechanical and adhesive properties.

As already reported for human cervical epithelial cells,^56^ the pericellular brush layer can be substantially different for normal and cancer cells. The bladder epithelial cells studied here revealed a similar pericellular brush structure, which is characterized by inner and outer layers (inner and outer brushes). Nonmalignant HCV29 cells are characterized by the shorter inner and longer outer brushes of various densities (the inner is denser than the outer brush) compared to the cancer TCCSUP cells. Further enzymatic treatment of the pericellular layer shows that only the outer brush responds to the treatment. It implies that the inner brush is mainly the corrugation of the pericellular membrane, which agrees with previously studied guinea pig cells.^23^

Distinct cell mechanics and adhesion accompany changes in the pericellular brush properties. Nonmalignant cells are stiffer than cancer cells and reveal a smaller nonspecific adhesion. The enzymatic treatment affects the adhesive properties of cells. Adhesion becomes even smaller for nonmalignant cells but larger for cancerous cells. In parallel, changes in the pericellular brush properties are observed in an enzyme-dependent manner (probably related to the location of the cleavage sites). The most abundant core proteins possessing HS are four members of the syndecan family^60^ that can potentially be used to identify bladder cancers.^36,61^ Syndecans are not the only HS-bearing molecules. Others include perlecan or glypicans.^62^ This and the effect of hep on HCV29 suggest that nonmalignant cells possess a higher level of HS chains that are not attached to syndecans. Simultaneously, the increased outer brush density after neu treatment in cancerous cells indicates a higher level of sialic acid residues, reported in the literature.^44^

## Conclusions

Here we studied the physical properties of bladder human epithelial cells with the help of the AFM indentation technique. The use of the brush model allowed us to study the mechanical properties of the cell body as well as the pericellular layer. Although it was previously reported that malignant cells were softer than nonmalignant, ignoring the pericellular layer, it substantially changes the derived values of the quasistatic Young’s modulus.^57^ Thus, it was interesting to verify if these changes stay when using the self-consistent brush model, which allows obtaining of the quasistatic Young’s modulus with an error of just a few percent.^24,25^ Here we observed that bladder epithelial cancer cells are still softer than nonmalignant ones, which qualitatively agrees with the previously reported observations.

The most nontrivial results of this work are in the study of the physical properties of the pericellular layer. We found a substantial difference in the structure and properties of the pericellular layer of both cell types, which demonstrated double-size brush behavior. The inner pericellular brush was longer for cancer cells, but its grafting density was similar to that found for nonmalignant cells. The outer brush was much shorter and less dense for cancer cells.

To understand the biochemical nature of the detected pericellular bush layer, we performed enzymatic treatment of cells with heparinase I and neuraminidase. This treatment showed significant effects only on the outer brush of the pericellular layer of both nonmalignant and cancerous cells. Neu affected mainly the cancerous cells by increasing substantially the effective grafting density of the outer brush. It implies that the number of sialic acid residues is substantially higher in the cancerous cells, which is in agreement with the previously reported data. Moreover, the increase of grafting density presumably indicates supramolecular restructuring of long polysaccharide molecules in a stiffer molecular construct. A substantial decrease of the outer brush was observed for both nonmalignant and cancerous cells after treatment with hep. It tells us that a major part of glycocalyx proteoglycans of the bladder epithelial cells contains heparan sulfate chains. These results show that subsequential treatment of cells with various enzymes applied to AFM experiments allows the detection of the molecular components and physical structure of the molecules of the pericellular layer. It is an important bridge between physics and molecular biology, by allowing translation of the measured physical properties of the pericellular layer into the language of biomolecules.

## Materials and Methods

### Cell Lines

Two human bladder cancer cell lines were used: nonmalignant cells of the ureter (HCV29, derived from the Institute of Experimental Therapy, PAN, Wroclaw, Poland) and transitional cell carcinoma (TCCSUP, ATCC, LGC Standards). HCV29 cells were cultured in RPMI-1640 medium (Sigma) supplemented with 10% fetal bovine serum (FBS, Sigma). TCCSUP cells were cultured in Eagle’s minimum essential medium (EMEM, LGC Standards) supplemented with 10% FBS. These different media represent the physiological conditions specific to each cell line. Cells were grown in culture flasks (Sarstedt) in an incubator (Nuaire) at 37 °C in 95% air and 5% CO_2_ and relative humidity above 98%. Cells were passaged when their confluence reached 80–90%. HCV29 and TCCSUP cells were detached from the surface using 0.05% and 0.25% trypsin–EDTA solution (Sigma) for 4 min, respectively. After a few passages, cells were seeded on a Petri dish (passages 6–8) for AFM measurements and on glass coverslips for fluorescence imaging.

### Enzymes

The stock solutions of heparinase I (hep, H2519 from Sigma, dissolved in 20 mM Tris-HCl, pH 7.5, 50 mM NaCl, 4 mM CaCl_2_, and 0.01% BSA) and neuraminidase (neu, N2876 from Sigma, dissolved in phosphate-buffered saline (PBS, Sigma)) were prepared at the concentration of 200 U/mL and 25 U/mL, respectively. The cells were initially cultured in the corresponding culture medium containing 10% FBS, then rinsed with 1% FBS and kept in it. Enzymes (hep or neu) were added to the cells for 1 h at the final concentration of 1 U/mL and 0.25 U/mL for hep and neu, respectively. After the treatment, cells were washed with PBS (PBS, Sigma) buffer, and the corresponding culture medium containing 1% FBS was added to a Petri dish.

### Determining G/F Actin Ratio

The G-actin/F-actin in vivo assay kit (catalog no. BK037, Cytoskeleton) was applied to quantify the ratio between G-actin and F-actin. Cells were homogenized in 300 μL of LAS2 buffer containing detergents, disrupting the cell membrane. As a result, only G-actin was solubilized, while F-actin was not. Next, the centrifuging of cell lysate at 100.000*g* at 37 °C for 1 h separated a soluble G-actin form from the polymerized one, F-actin. F-actin was present as a pellet located at the bottom of the Eppendorf tube, while the supernatant contained monomeric G-actin. Next, Western blot was applied to analyze the G- and F-actin contents. Briefly, the collected supernatants were separated on 10% SDS–PAGE gels and transferred to a PVDF membrane. Anti-actin rabbit polyclonal antibody (Cytoskeleton) was applied to detect G- and F-actin. Bands were visualized using horseradish peroxidase-coupled secondary anti-rabbit antibody (Cell Signaling Technology). The ratio between G/F actin was calculated using ImageJ software based on the densitometry approach.

### Confocal Microscopy

Cultured on the surface of the chambered coverslips placed in a Petri dish (μ-Slide 18 Well, Ibidi), the cells were fixed with 3.7% paraformaldehyde in PBS for 20 min at room temperature (RT). Next, they were washed with PBS (3 times for 2 min), treated with 0.2% cold Triton X-100 in PBS for 4 min at 4 °C, and washed again with PBS. Next, the cells were incubated for 30 min at RT with phalloidin conjugated with Alexa Fluor 488 (1:200 in PBS). Next, the cells were washed in PBS and incubated with Hoechst 33342 dye (1:5000 in PBS; used to visualize the cell nucleus) for 15 min at RT. After rinsing with PBS, cells were kept in PBS for confocal imaging. Images of actin filaments were acquired using a confocal microscope (Leica TCS SP8 WLL) equipped with new-generation HyD detectors. Fluorescent dyes were excited at 405 nm (Hoechst 33342 via UV CW laser) and 499 nm (Alexa Fluor 488 via WLL white laser). Images were acquired using an oil immersion 63× objective lens (HC PL APO CS2 NA 1.40).

### AFM Cantilevers

MLCT-SPH-DC silicon nitride cantilevers (Bruker-Nano, CA, USA) were chosen. Each cantilever was precalibrated by the manufacturer using a laser Doppler vibrometer. The fact that each cantilever was independently calibrated allows us to know the real cantilever spring constant used in the experiments and apply the SNAP protocol^63^ to calibrate the sensitivity of the photodetector. The resonance frequency of the used cantilevers ranges from 6 to 12 kHz, corresponding to a range of spring constants from 12 to 50 mN/m. At the free end of each cantilever, a hemispherical probe, i.e., a cylinder ended with a half spheroid with a radius of 5 μm, was mounted.

### AFM Measurements

The AFM-based indentation measurements were conducted on the top of the cell, i.e., over the whole cell. Each cell was mapped with a grid of 15 pixels ×15 pixels set on each map (scan size from 50 μm × 50 μm to 70 μm × 70 μm). At each point, an individual force curve was recorded (i.e., the relation between the cantilever deflection and the relative sample position). The relative sample position was linearly changed at a speed of 6 μm/s. The maximum load force was set to 7–8 nN. The cell geometry was saved with collection of the force curves. The latter allowed one to calculate the cell radius.^57^

### Statistical Significance

Statistical significance was determined based on Kruskal–Wallis ANOVA nonparametric test using the confidence level starting with *p* < 0.05.

### Mechanical Properties of Cells: The Brush Model

If one wants to use the concept of elastic modulus, it has to be consistent with the definition of such a modulus; i.e., the material should be homogeneous and isotropic. Although a cell can hardly be treated as a homogeneous material, it can be a good approximation for relatively small deformations. As was shown previously, the approximation of homogeneity works well if and only if (1) one takes into account the presence of an essentially nonlinear pericellular brush layer and (2) one uses a relatively dull AFM probe.^24^ This approach is called the brush model. It was demonstrated that this model unambiguously separated the elastic properties of the cell body from the force signature of the pericellular brush layer, allowing self-consistent calculations of the quasistatic (elastic) Young’s modulus and, in addition, the properties of the pericellular brush.^64^ Later the model was further elaborated. The most comprehensive analysis of the model was done recently to evaluate its robustness, i.e., a weak dependence of the model results in possible uncertainties in the model itself and ambiguities in the experimental data^24,25,65^

**Figure 10:**
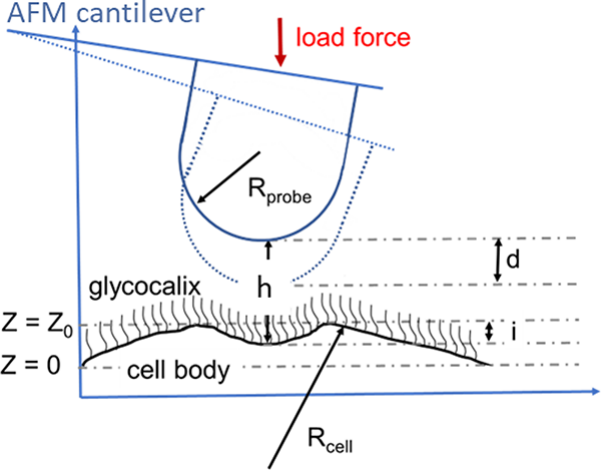
Schematic of interaction between an AFM spherical indenter (probe) and cell demonstrating definitions of the parameters used in the brush model. *Z* is the vertical position of the AFM scanner, *d* is the cantilever deflection, *Z*_0_ is the undeformed position of the cell body, *i* is the deformation of the cell body, *Z* = 0 is at the maximum deflection (assigned by the AFM user), and *h* is the separation between the cell body and AFM probe.

The brush model is described in detail in the above references. Here we briefly describe the major steps used to process the AFM force curves through this model. Figure 10 shows a schematic representation of an AFM probe deforming the surface of a cell surrounded by the pericellular brush layer.

The following equation describes the distance between the cell body and the spherical indenter:
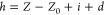
1where *Z*_0_ is the position of the undeformed cell body, *h* is the distance between the AFM probe and the surface of the cell body, and *i* is the deformation of the cell body. The latter can be calculated using the hertz model:
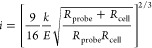
2where *E* is the quasistatic Young’s modulus, *k* is the spring constant of the AFM cantilever, and *R*_probe_ and *R*_cell_ are the radii of the AFM probe and cell, respectively. The Poisson ratio of a cell is chosen to be 0.5. Assuming that the PB layer is softer than the cell, at the maximum indentation the brush layer is almost squeezed (*h* = 0), and the modulus of the cell body is calculated by fitting the indentation curve within the squeezed brush region.

The glass slide on which the cells are sitting is a rigid substance, which can overestimate the quasistatic Young’s modulus. The effect was described in the literature.^66,67^ The following correction is used for the hertz model:
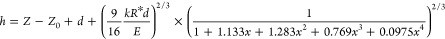
3where *Z* represents the relative vertical scanner position of the cantilever, *d* is the cantilever deflection, *Z*_0_ is the undeformed position of the cell body, *h* is the separation between the cell body and AFM probe, *E* is the elastic modulus of the cell body, *k* is the cantilever spring constant, and
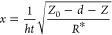
4where *h*_*t*_ is the cell height. The effective radius, R*, is defined as 
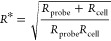
5where *R*_probe_ and *R*_cell_ are the radii of curvature of the AFM probe and cell, respectively.

A clear exponential dependence, a characteristic feature of the force due to the brush layer, agrees with a molecular brush described by the Alexander–de Gennes model^68,69^ for an entropic polymer brush:
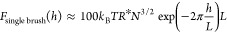
6where *k*_B_ is the Boltzmann constant, *T* is the temperature, *N* is the surface density of the brush constituents (grafting density or effective molecular density), and *L* is the equilibrium thickness of the brush layer. Note that this formula is valid provided 0.1< *h*/*L* < 0.8. In the case that the indentation curve demonstrated a double slope behavior in logarithmic scale, the longer and softer brush and the shorter and more rigid brush can be described by a simple sum of two brush forces:^64^
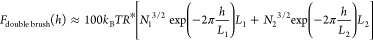
7where *N*_1_, *L*_1_, and *N*_2_, *L*_2_ are the parameters of the larger and smaller brush, respectively.

### Work of Adhesion

The retraction part of the force curves was analyzed to determine the work of adhesion, i.e., the work needed to detach a bare hemispherical probe from the cell surface. Developing even nonspecific adhesion between nonfunctionalized silicon nitride hemispherical probe and cell surface typically requires a much longer time than the duration of the contact during the described measurements.^70,71^ Therefore, the observed pull-off force is presumably a combination of a viscous hydrodynamic interaction between the AFM probe and cell surface. Electrostatic forces could also quickly form a bond, but they are not expected to be prevalent because of the negatively charged silicon nitride surface and mainly negatively charged molecules on the cell surface. The work of adhesion was calculated as the area included within the adhesive part of the force curve.

## Supporting Information Available

Figures S1 and S2 showing examples of the brush model analysis for single (Figure S1) and double (Figure S2) behavior for a nonmalignant HCV29 and cancerous TCCSUP cells; Figure S3 showing cleavage sites for neuraminidase and heparinase I; Figure S4 showing fluorescently labeled F-actin in a nonmalignant HCV29 and cancerous TCCSUP cells before and after enzymatic treatment; Figures S5 and S6 showing the distribution of the work of adhesion in a nonmalignant HCV29 and cancerous TCCSUP cells before and after enzymatic treatment; Figure S7 showing the way of cell height determination from the AFM data (PDF)
