# Supplementary material for: Mechanical Way To Study Molecular Structure of Pericellular Layer
Source: ACS Appl Mater Interfaces. 2023 Jul 25;15(30):35962–72. doi: 10.1021/acsami.3c06341 (PMC10401571; doi:10.1021/acsami.3c06341)
Supplement: Supplementary file 1 — am3c06341_si_001.pdf [file am3c06341_si_001.pdf]

# Supporting Information

# Mechanical way to study molecular structure of pericellular layer

*Nadezda Makarova[1], Małgorzata Lekka[2*], Kajangi Gnanachandran[2], Igor Sokolov[1,3*]*

[1] Department of Mechanical Engineering, Tufts University, Medford, MA 02155, USA

[2] Department of Biophysical Microstructures, Institute of Nuclear Physics PAN, PL-31342 Kraków, Poland

[3] Department of Physics, Tufts University, Medford, MA 02155, USA

Corresponding authors:

malgorzata.lekka@ifj.edu.pl

Igor.Sokolov@tufts.edu

ORCID IDs:

| | |
|---|---|
| Malgorzata Lekka | 0000-0003-0844-8662 |
| Kajangi Gnanachandran | 0000-0003-4874-3842 |
| Igor Sokolov | 0000-0001-6260-4326 |

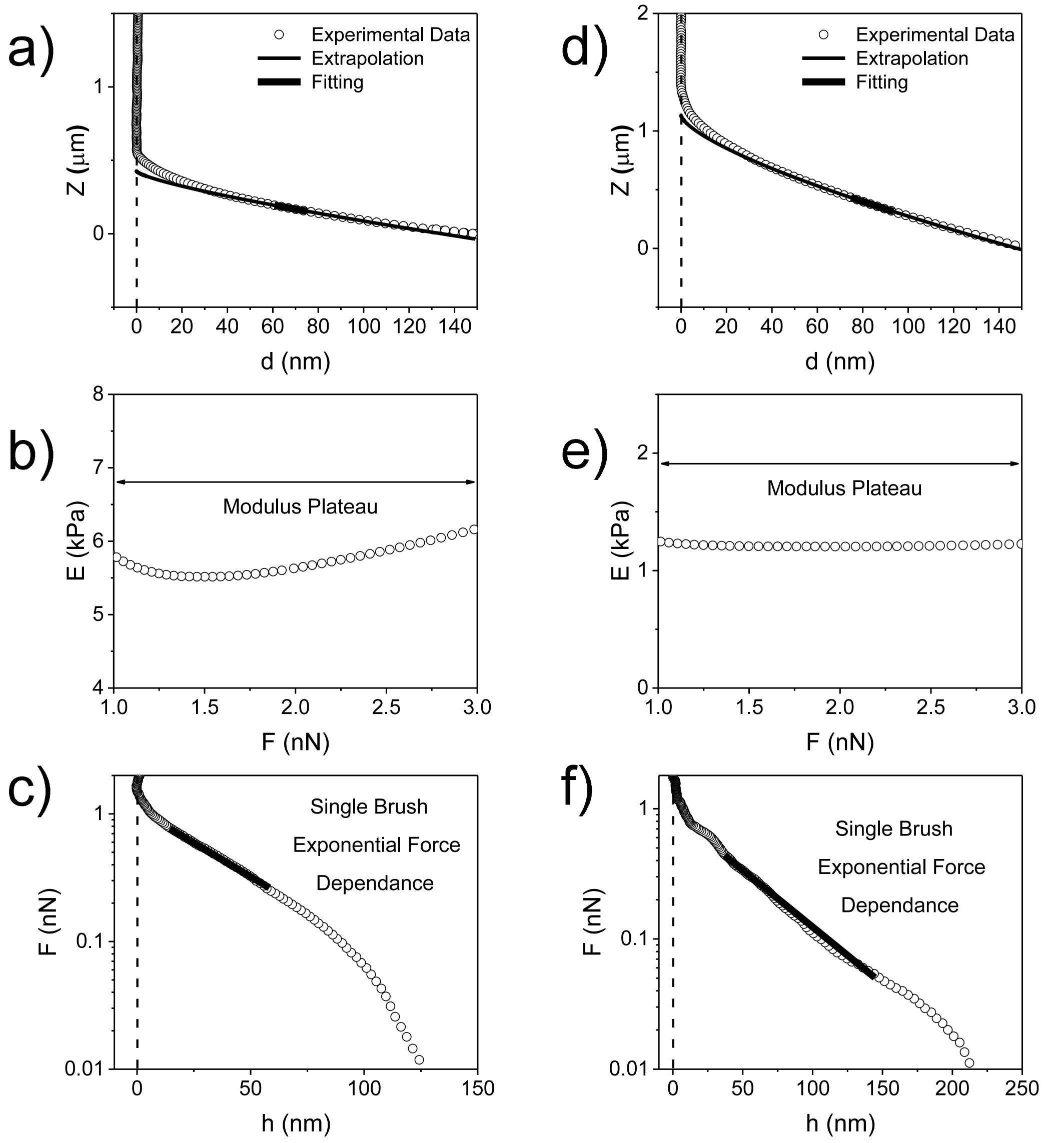


Figure S1. An example of the brush model analysis for single brush behavior for (a-c) a nonmalignant HCV29 and (d-f) cancerous TCCSUP cells. (a and d) raw data, the dependence of the deflection of the cantilever d on the vertical position of the sample Z. (b and e) the dependence of the quasistatic Young's modulus on the load force, where the modulus corresponding to the plateau is treated as the modulus of the cell body. The specific examples give the valley of the modulus as $E = 5.5$ kPa for nonmalignant and E = 1.2 kPa for the cancer cell. Note: this dependence is for the case of fixed $Z_0$. (c and f) The force is due to the brush. This is an example of a single brush, with the fitted brush parameters L = 210 nm, N = 290 $1/\mu m^2$ for nonmalignant and L = 260 nm, and N = 280 $1/\mu m^2$ for the cancer cells (this figure is also included in the PhD thesis of Nadezda Makarova "*Advanced Experimental and Theoretical Methods to Study Nano Mechanics of Soft Materials*." Order No. 30245595, Tufts University, 2023, USA).

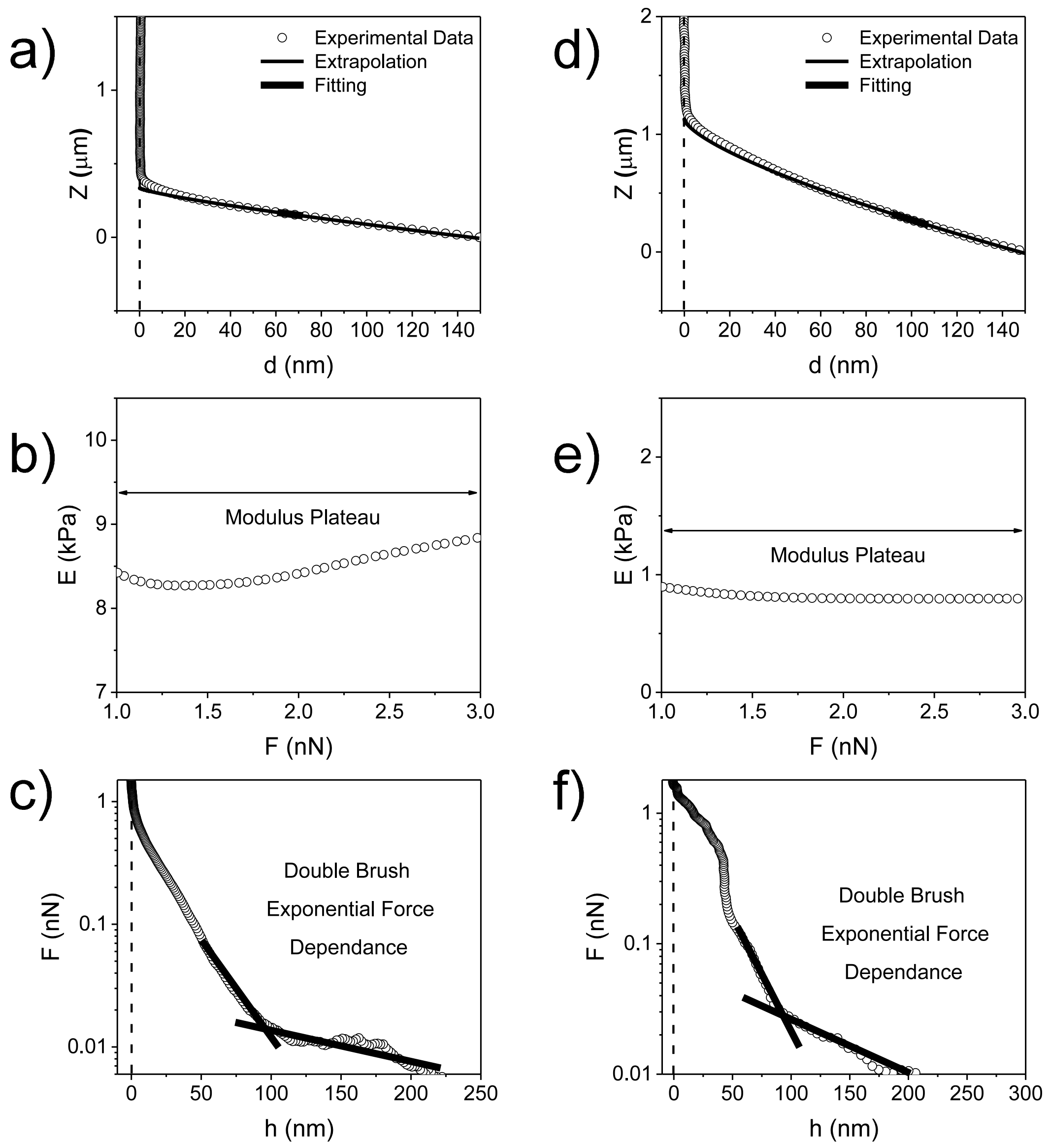


Figure S2. An example of the brush model analysis for double brush behavior for (a-c) nonmalignant HCV29 and (d-f) cancerous TCCSUP cells. (a and d) raw data, the dependence of the deflection of the cantilever d on the vertical position of the sample Z. (b and e) the dependence of the quasistatic Young's modulus on the load force, where the modulus corresponding to the plateau is treated as the modulus of the cell body. The specific examples give the valley of the modulus as $E$ = 8.3 kPa for nonmalignant and E = 0.80 kPa for the cancer cell. Note: this dependence is for the case of fixed $Z_0$. (c and f) The force is due to the brush. This is an example of double brush, with the fitted brush parameters $L_1$ = 130 nm, $N_1$ = 190 $1/\mu m^2$ and $L_2$ = 3300 nm, $N_2$ = 1.4 $1/\mu m^2$ for nonmalignant and $L_1$ = 140 nm, $N_1$ = 330 $1/\mu m^2$ and $L_2$ = 2300 nm, $N_2$ = 2.7 $1/\mu m^2$ for the cancer cells this figure is also included in the PhD thesis of Nadezda Makarova "*Advanced Experimental and Theoretical Methods to Study Nano Mechanics of Soft Materials*." Order No. 30245595, Tufts University, 2023, USA).

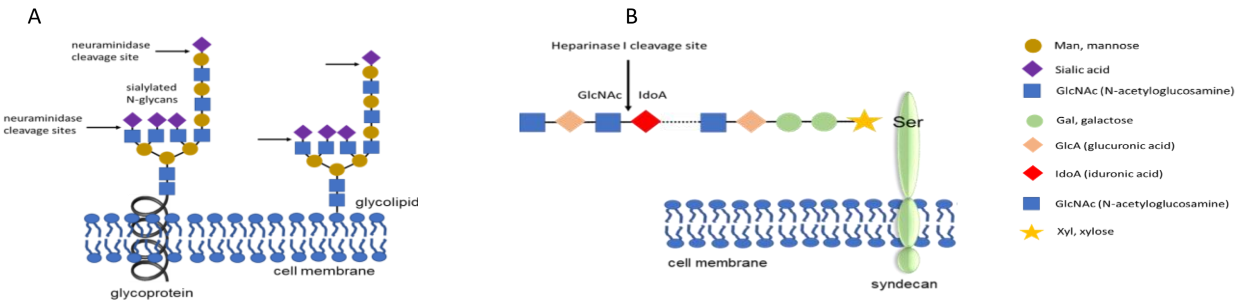


Figure S3. (A) An example of sialylated N-glycans with sialic acid residues at the end of each branch. These sialic acid residues are the target for neuraminidase. (B) Exemplary heparan sulfate chains attached to syndecan (a protein) embedded in the cell membrane, with indicated heparinase cleavage site.

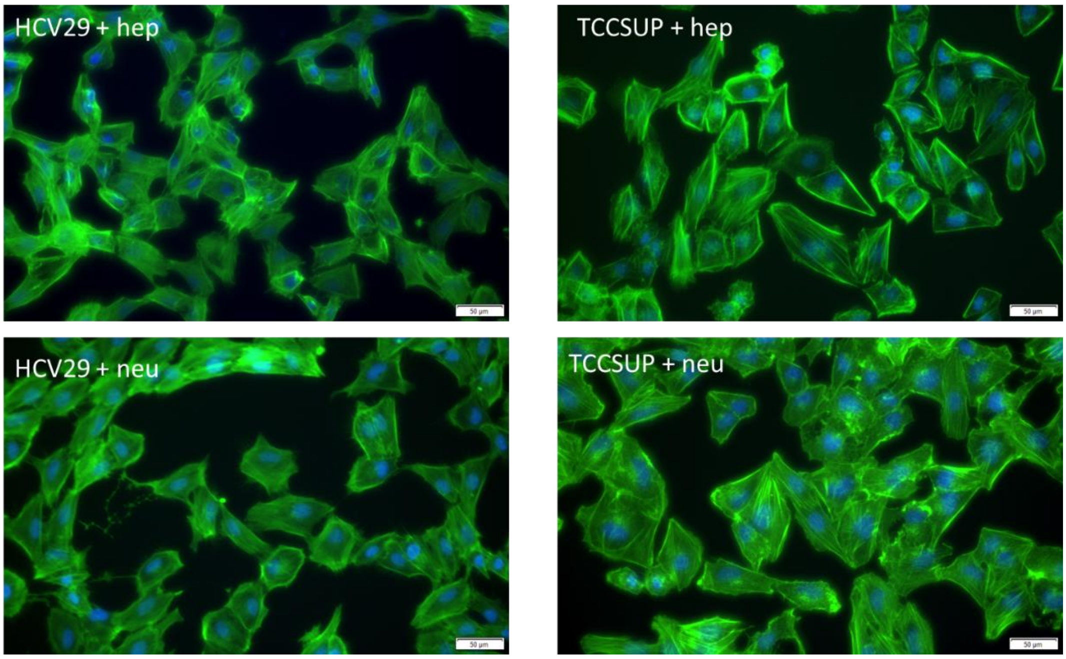


Figure S4. (A) Fluorescent images of the actin cytoskeleton in HCV29 and TCCSUP cells treated with enzymes (Hep and Neu). Staining: blue–cell nucleus (Hoechst); green–actin filaments (phalloidin conjugated with Alexa Fluor 488). Scale bar 50 um.

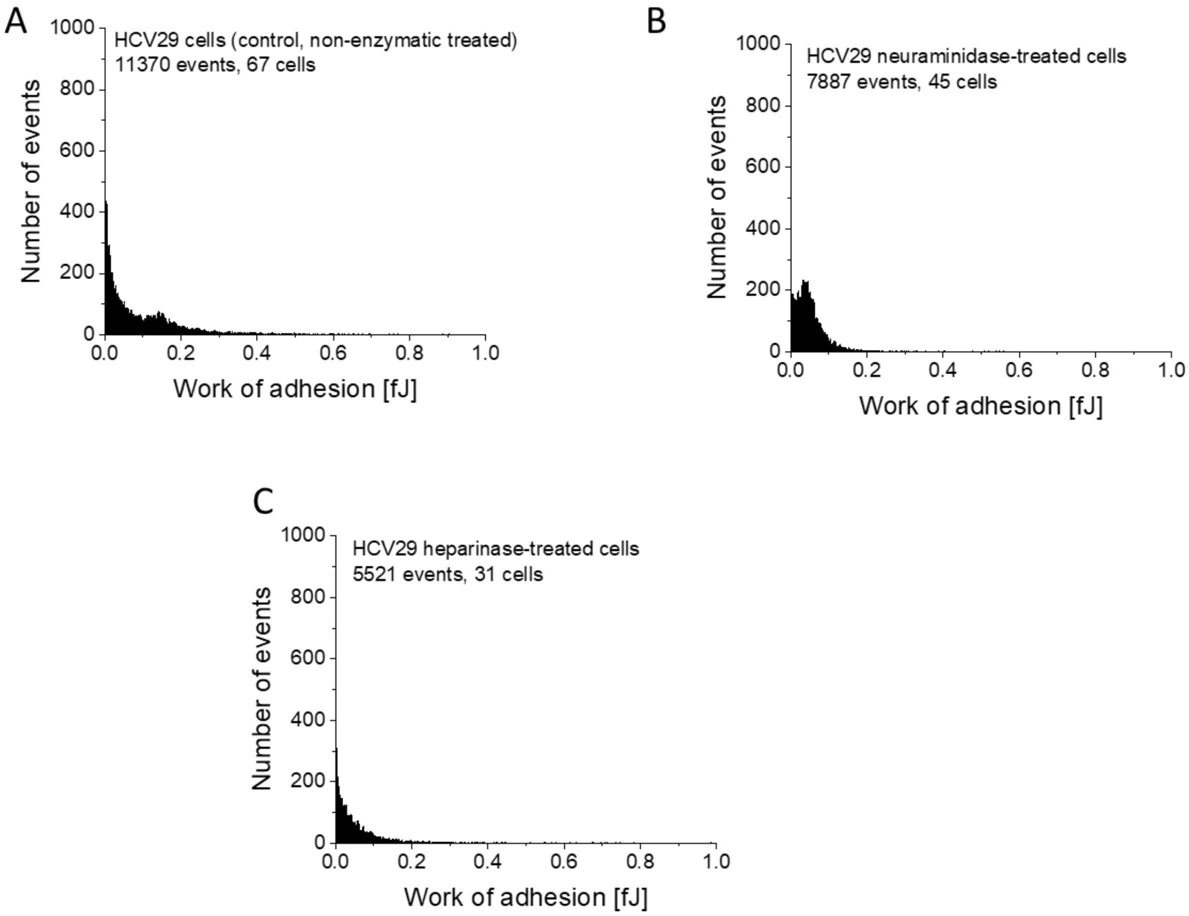


Figure S5. Work of adhesion determined for nonmalignant HCV29 cells: (A) control, non-enzymatic treated, (B) neuraminidase-treated, and (C) heparinase-treated cells.

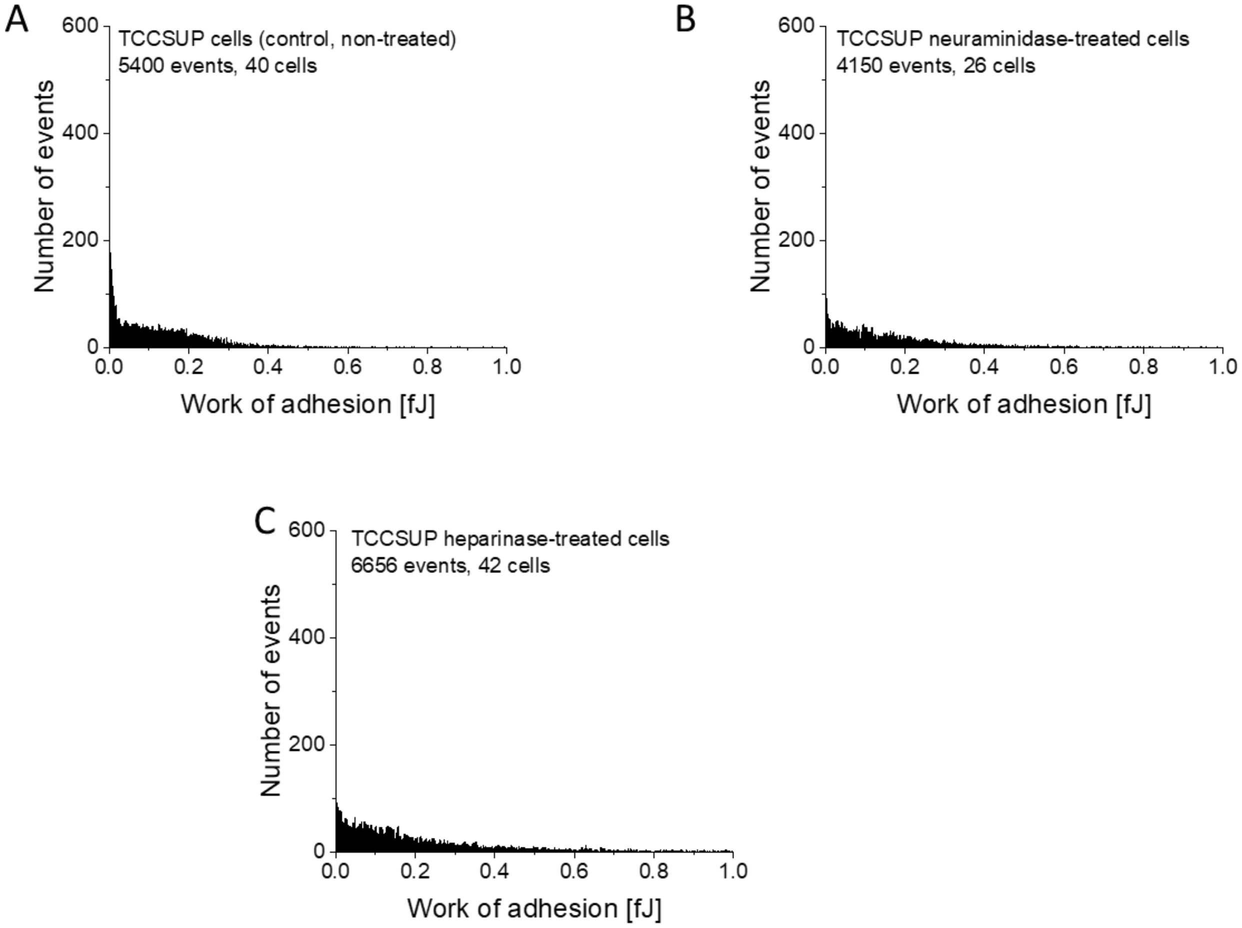


Figure S6. Work of adhesion determined for cancer TCCSUP cells: (A) control, non-enzymatic treated, (B) neuraminidase-treated, and (C) heparinase-treated cells.

## Cell height determination

Cell height was determined from the contact point image of a single and surrounding substrate (at the contact point image, the load force is assumed to be zero, Fig. S7A). Then, a cross-section along one direction was plotted, to which a Gauss function was fitted (Fig. S7B). The maximum of the fitted function corresponds to the cell height.

A

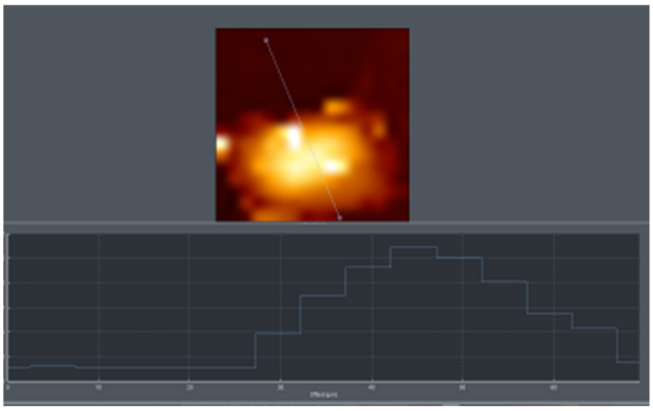

B

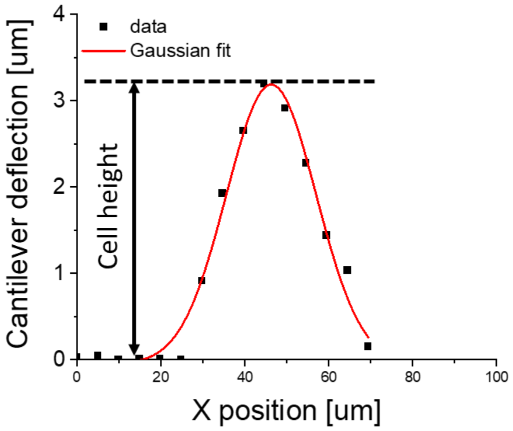


Figure S7. (A) Exemplary image of a single cell (contact point position) with a cross-section represented as a histogram (below), scan size 50 µm. (B) Cross-section through the cells and substrate (black dots), to which a gaussian function was fitted. The maximum corresponds to the cell height. It should be noted that in the case of spherical probes, surface glycocalyx or membrane corrugations may influence the determined cell height because the cantilever deflection reflects the interaction between the probe and the cell surface. In this parcticular case, the cell height was determined to be 3.2 µm.
